# Exploring the mediating effects of negative and positive religious coping between resilience and mental well-being

**DOI:** 10.3389/fnbeh.2022.954382

**Published:** 2022-10-20

**Authors:** Janusz Surzykiewicz, Sebastian Binyamin Skalski, Małgorzata Niesiobędzka, Karol Konaszewski

**Affiliations:** ^1^Faculty of Philosophy and Education, Katholische Universität Eichstätt-Ingolstadt, Eichstätt, Germany; ^2^Faculty of Education, Cardinal Stefan Wyszynski University in Warsaw, Warsaw, Poland; ^3^Faculty of Education, University of Białystok, Białystok, Poland

**Keywords:** wellbeing, resilience, religious coping, catholics, mental health

## Abstract

**Background:**

The purpose of the study was to examine more thoroughly the relationship between trait resilience and mental well-being. Although research demonstrates that this relationship is partially mediated by stress-related variables, no study has taken into account the mediating role of religious coping. We examined the mediating role of both variants of religious coping, positive and negative, along with specific strategies within the scope of religious coping strategies in a group of practicing Catholics.

**Method:**

Participants were 317 people aged 19–60 years (*M* = 24.34; *SD* = 6.30). The respondents indicated their gender and age, and then completed the RS-14 (trait resilience), RCOPE (religious coping), and WEMWBS (mental well-being) scales.

**Results:**

The results displayed a significant relationship between resilience and mental well-being (*r* = 0.67; *p* < 0.001). The relationship between resilience and positive religious coping was negligible (*r* = 0.09; *p* = 0.74), contrary to the relationship between resilience and negative coping that was significant but weak (*r* = −0.29; *p* < 0.001). Although the relationships between overall negative and positive religious coping with mental well-being were irrelevant, we found significant relationships between some strategies and mental well-being. The mediation analysis has demonstrated that the general negative religious coping and the strategies of demonic reappraisal, passive religious deferral, and spiritual discontent have enhanced the positive relationship between resilience and mental well-being. Contrary to expectation, positive strategies did not mediate the relationship between resilience and mental well-being, except religious practices (*c*^′^ *path* totaled *β* = 0.66; *t* = 15.74, *p* < 0.001). The insignificant mediation effect can stem from the fact that the relationship between positive religious coping and stress is noticeable only in the long term. We controlled age and sex as statistically significant covariates so that the mediation effects obtained were devoid of the influence of those critical variables on the models.

**Conclusion:**

This is the first study to investigate the role of religious coping as a mediator in the relationship between resilience and mental well-being.

## Introduction

For over three decades, numerous studies have shown that the trait of resilience significantly connects with broadly understood mental health and well-being. Research demonstrates that resilience relates negatively to the level of perceived stress, anger, hostility, generalized anxiety, depression, post-traumatic stress, and mental disorders (Beasley et al., 2003; Agaibi and Wilson, 2005; Campbell-Sills et al., 2006; Catalano et al., 2011; Ya et al., 2012; Wu et al., 2013; Surzykiewicz et al., 2019; Konaszewski et al., 2021a; Tamura et al., 2021) and positively to mental well-being, satisfaction with life, quality of life, positive adaptation, as well as physical and mental health (Armstrong et al., 2005; Friedli, 2009; Liu et al., 2013; Collishaw et al., 2016; Cosco et al., 2017). Research focused on exploring the relationship between resilience and well-being demonstrates that this relationship is at least partially mediated by perceived stress (Park et al., 2018), perceived stressors (Johnson et al., 2019), coping styles (Chen, 2016), stress-coping strategies (Kaczmarek et al., 2011), sense of social support (Zhang et al., 2017), hope (Satici, 2016), self-compassion (Kotera et al., 2020), Internet addiction (Mak et al., 2018), and depression (Lu et al., 2017). Among the studied mediators, stress-related variables are dominant, but at the same time, no study has taken into account the mediating role of religious coping. Our research aims to fill this gap. In this study, we extend the scope of our findings and analyze the potential mechanism linking the resilience feature with mental well-being, pointing to the mediating role of religious coping.

Religion constitutes an integral part of the lives of many people around the world (Zhang et al., 2017) and has a beneficial effect on their personal adaptation (Zarzycka and Zietek, 2019). A growing number of studies suggests that people often turn to various aspects of religion in stressful situations (Zinnbauer and Pargament, 1998; Koenig, 2009; Cassibba et al., 2014; Formoso-Suárez et al., 2022), in order to maintain a sense of control (Sasaki and Kim, 2011), regain mental balance after experiencing stress (Zinnbauer and Pargament, 1998), find meaning in life (Pargament, 1997) and maintain appropriate social relations (Páez et al., 2018). Research also demonstrates that people with high religious and spiritual commitment assess their lives more positively, despite all possible negative circumstances (Koenig, 2012, 2020). They possess a higher level of well-being and display lower scores within the sphere of perceived stress (Ramsay et al., 2019; Vishkin et al., 2019; Saud et al., 2020). Religion can also be a source of distress. An example in this area is religious struggles, which are described from the perspective of religious stress coping theory (Pargament et al., 2000, 2005). Religious struggles refer to forms of distress and conflict that involve religious and spiritual realities (Zinnbauer et al., 1999). Struggles occur when certain aspects of a person’s current belief system, practices, or experiences become the center of negative thoughts and emotions, worry, or conflict (Exline, 2013; Exline et al., 2014). Research has shown that religious struggles and tensions negatively correlated with well-being, life satisfaction, quality of life, self-esteem, coping resources, internal locus of control, optimism, gratitude, prosocial sensitivity, and prosocial behavior (Koenig, 2012, 2020; Lucchetti and Lucchetti, 2014; Abdel-Khalek, 2019; Lucchetti et al., 2021; Konaszewski et al., 2022). In our opinion, in the context of the presented research results, it is worth taking a closer look at both the direct religious relationship between coping with mental well-being and the indirect role of religious coping in the relationship between resilience and mental well-being.

### The holistic perspective on mental well-being

Research pertaining to psychological well-being highlights two research directions that can be distinguished, on the basis of theoretical assumptions and philosophical traditions: one concerns human happiness (hedonistic perspective; see e.g., Ryan and Deci, 2001), while the second is related to human potential (eudaimonic perspective; see e.g., Ryff and Singer, 1998). Moreover, numerous studies indicate that mental well-being is a multidimensional phenomenon that combines hedonistic and eudaimonic aspects (Stewart-Brown, 2013, 2016). In hedonistic terms, well-being consists of subjective happiness and refers to the experience of pleasure rather than dissatisfaction, including all judgments regarding the good and bad elements of life. This means that happiness cannot be reduced to physical hedonism, because it can be gained by achieving goals or worthwhile results in various fields (Diener et al., 1998; Diener, 2000). On the other hand, in the eudaimonic approach, the essence of well-being is associated with the idea of living in harmony with oneself. Eudaimonic well-being goes beyond the sense of pleasure in the life of an individual and encompasses a higher degree of psychosocial integration, broadly understood as a “good life” (Waterman, 1993). In turn, the holistic perspective assumes that mental well-being includes hedonistic (positive feelings, affects, emotions) and eudaimonic (positive functioning, mentality and relationships) dimensions (Stewart-Brown, 2013, 2016; Lyu et al., 2021). The hedonistic dimension relates to feelings, i.e., emotional well-being, and manifests itself, for example, in the form of positive and negative affects and satisfaction with life. Feelings are viewed as a state of mind that can vary depending on the situation, which is often beyond the control of an individual (Stewart-Brown, 2016). The eudaimonic dimension is related to the functioning of an individual, both on a personal and social level (e.g., mental or social well-being). This type of well-being is achieved through the development of character traits and behaviors (Stewart-Brown, 2013, 2016).

### Different concepts of resilience

Many life events affect well-being (Luhmann et al., 2012). Personality traits also have a significant impact on the level of well-being. A meta-analysis of research results demonstrated that neuroticism is most strongly related with well-being. Other traits, related to a lesser extent, include repressiveness, trust, emotional stability, locus of control, hardness, positive affect and self-esteem (DeNeve and Cooper, 1998). Resilience is also a trait linked to well-being. Resilience is described as an umbrella term, the symbolism of which expresses well the entirety of theoretical and research approaches in this area (Konaszewski, 2020). Depending on how it is described, explained, and delimited, different concepts of resilience can be distinguished: resilience as a trait, as a process, as a capability, or as an outcome. Defined as a dynamic process, resilience reflects relatively good adaptation, i.e., the positive adaptation of an individual despite the threats, adversities, or traumas they experience. This process involves the interplay of a spectrum of risk factors, vulnerabilities, and protective factors (Luthar et al., 2000; Masten, 2001; Wright and Masten, 2005; Windle et al., 2008; Windle, 2011). Resilience has also been defined as the ability to bounce back or recover from stress, to adapt to stressful circumstances, to not become ill despite significant adversity, and to function above the norm in spite of stress or adversity (Smith et al., 2008). In turn, Bonanno et al. (2011) view resilience as a pattern of outcomes after potentially traumatic events characterized by a stable trajectory of healthy mental and physical functioning. In this approach, resilience is the manifestation of emotional, behavioral, or health outcomes that meet or exceed normative developmental milestones, behavioral functioning, or emotional well-being despite exposure to significant life challenges (Hilliard et al., 2015).

### Relationships between resilience as a personality trait and mental well-being

In our study resilience, understood as a personality trait, alleviates the negative effects of stress, promotes the ability to cope with changes or adversities and supports adaptation in difficult situations. Persons with high levels of resilience are able to adapt to overwhelming adversities and restore balance to life, avoiding the potentially harmful effects of stress. The strength of resilience is influenced by life circumstances and interventions undertaken (Wagnild and Young, 1993). Studies have demonstrated a significant positive relationship between resilience and both hedonistic and eudaimonistic well-being (Wagnild and Young, 1993; Abolghasemi and Varaniyab, 2010; Windle, 2011; Ya et al., 2012; He et al., 2013; Liu et al., 2013; Aiena et al., 2015; Di Fabio and Palazzeschi, 2015; Smith and Hollinger-Smith, 2015; Surzykiewicz et al., 2019). Moreover, resilience affects well-being not only directly but also indirectly through its impact on the ability to cope with stress. It makes it easier to mobilize oneself to take remedial actions in stressful situations.

### Relationships between resilience as a personality trait and coping

Carver et al. (1989) emphasize the significant influence of resilience on coping; it determines the selection of specific coping strategies. Research demonstrates that the higher the resilience level, the wider the range of applied strategies (Bogar and Hulse-Killacky, 2006) and the greater the propensity to use problem-focused strategies and the lower the likeliness of using strategies focused on negative emotions and the need to discharge them (Boyden and Mann, 2005; Campbell-Sills et al., 2006; Chen, 2016; Konaszewski et al., 2019). As has already been indicated, research confirms the mediating role of coping in the relationship between resilience and well-being (Sojo and Guarino, 2011; Malkoç and Yalçin, 2015). As far as our knowledge goes, there have been no studies analyzing the mediating role of one of the important, especially in the case of believers and practitioners, ways of coping with difficult life situations, i.e., religious coping. This is why we believe it is worth exploring the relationships connecting religious coping, resilience, and well-being.

### Positive and negative religious coping

In the last three decades, in terms of searching for sources of inner strength in difficult situations, theoretical concepts and scientific studies have confirmed the special role of religious coping (Pargament et al., 1990, 2000, 2001; VanderWeele, 2017; Weinberger-Litman et al., 2020). Religious coping is a multidimensional construct, with positive and negative aspects (Ano and Vasconcelles, 2005). Religious coping involves a number of cognitive and behavioral techniques aiding the individual to cope with or adapt to difficult life situations (Pargament et al., 2013). Positive religious coping is associated with the positive commitment of individual forces in the sphere of religion. Examples of positive coping include seeking religious and spiritual support, positive religious judgment, and the individual’s interactions with God. The negative religious coping pattern manifests itself, *inter alia*, in dissatisfaction with God and the religious community, or negative feelings towards a given event, perceived as God’s punishment or as the actions of the devil (Krok, 2014, 2015).

### Relationships between positive and negative religious coping and mental well-being

An intensified stream of research on adaptive resources has emerged recently, with positive and negative religious coping having been recognized as important predictors of contentment, life satisfaction and quality of life (Koenig et al., 2012; Koenig, 2018; Oman, 2018). Numerous studies have also confirmed that religious coping is an important predictor of well-being (Burker et al., 2004; Rippentrop et al., 2005; Zwingmann et al., 2006; Trevino et al., 2010; Hawkes et al., 2021). The results obtained regarding the relationship of positive religious coping with well-being are less unambiguous than data demonstrating the mutual relationship between well-being and negative religious coping. Certain studies have demonstrated that positive religious coping is linked with a high level of well-being (Pargament et al., 2001; Cole, 2005; Wnuk, 2007; Scandrett and Mitchell, 2009). However, in other studies, the relationship between the two variables proved to be insignificant (Fitchett et al., 1999; Hebert et al., 2009; Krok, 2014). The data on negative religious coping and well-being are much more consistent. Negative religious coping has been linked with decreased quality of life, including poorer physical and social functioning, vitality and mental health (Pargament et al., 2001; Wnuk, 2007; Hebert et al., 2009; Scandrett and Mitchell, 2009; Krok, 2014; Taheri-Kharameh et al., 2016). Moreover, studies also show that particular categories of religious struggle and tensions are linked with high emotional distress, poorer indicators of health, and lower quality of life and well-being, both in the normal population and in various clinical trials (Exline, 2013; Zarzycka, 2017). Studies have demonstrated that religious doubts negatively correlated with well-being, with the effect stronger in younger people than in older people (Krause et al., 1999). A similar negative relationship pattern was observed between divine, demonic struggle, moral struggle, ultimate meaning struggle and well-being (Abu-Raiya et al., 2015; Zarzycka and Puchalska-Wasyl, 2019). Only certain studies presented the relationship between demonic struggle and well-being as insignificant and the relationship between moral struggle and well-being as positive (Zarzycka et al., 2020). And so, adjustment to specific religious and spiritual struggles may have a distinct role in predicting satisfaction with life and well-being (Wilt et al., 2016). It can therefore be concluded that the results of research in this area indicate a clearly defined negative relationship between well-being and negative religious coping. In contrast, relationships between positive religious coping and well-being are less consistent. This is why it is worth taking a closer look at the relationships between multidimensional well-being and individual strategies involved in positive and negative religious coping.

### Purpose of the study

The purpose of the study was to gain insight into reciprocal relationships between trait resilience and mental well-being. We conducted mediation analysis due to the prominent role of mediating variables in psychological theory and research. Mediation analysis is considered an essential research tool, and it *“…is now almost mandatory for new social-psychology manuscripts”* (Bullock et al., 2010, p. 550). Although research demonstrates that the relationship between resilience and mental well-being is partially mediated by stress-related variables, no study has taken into account the mediating role of religious coping. Our research aims to fill this gap. In the study, we examine the mediating role of positive and negative religious coping in the relationship between trait resilience and mental well-being. Religious coping is remarkably crucial for practicing Catholics. Research on well-being and religious aspects is undertaken quite often in our times, but it does not take into account mental well-being as a whole. In line with the literature review presented above, a conclusion can be drawn that the research to date presents well-being in either hedonistic or eudaimonic terms. By focusing on a single dimension of well-being, research fails to yield conclusive results. This is why, in our opinion, it is worth introducing a multidimensional approach to well-being, combining hedonistic and eudaimonic aspects (Stewart-Brown, 2013, 2016), in order to describe the relationship between resilience, mental well-being, and religious coping in more detail. Furthermore, in the study, we analyze the mediating role of both variants of religious coping, i.e., positive and negative, along with specific strategies within the scope of religious coping strategies in a group of practising Catholics.

## Method

### Participants and procedure

The study was carried out in the winter of 2020 in Poland during the second wave of the COVID-19 pandemic, with the consent of the university ethics committee. Participants were 317 people aged 19–60 years (*M* = 24.34; *SD* = 6.30), including 75% women. Selecting the sample was purposeful. The research covered people identifying themselves as practising Catholics. No additional recruitment criteria were required. The invitation to participate in this study was sent using the academic websites of the University of Bialystok and Cardinal Stefan Wyszyński University in Warsaw. The link was sent to full-time and part-time students. The link to the survey was active from December 1 to December 20. The Google Forms platform was used to collect data. Participants who expressed their willingness to take part in the study received a link to the online questionnaire, with an access password. Each participant provided their informed consent to anonymous participation in the study. After giving consent, the respondents indicated their gender and age, and then completed the RS-14 (trait resilience), RCOPE (religious coping), and WEMWBS (mental well-being) scales. All procedures performed in the study involving human participants were under the ethical standards of the Ethics Committee of the University of Bialystok in Bialystok. The survey was anonymous and did not require providing any personal data. More details are presented in Table 1.

**Table 1:** Baseline characteristics of respondents.

Study groups		Women	Men
Sex	N (%)	237 (75)	80 (25)
Age	Mean (SD)	24.35 (6.75)	24.30 (4.73)
	Range	19–60	19–50
Country participating in the study		Poland	

### Research tools

#### Trait resilience

For the measurement of *resilience*, understood as a personality trait, the *Resilience* Scale 14 (RS-14) by Wagnild and Young (1993) was used. This tool consists of 14 statements. Participants are asked to respond to each of them using a 7-point scale from 1: I disagree to 7: I agree. The distribution of points within the RS-14 scale is in the area of 14–98. Polish adaptation studies validated the original one-dimensional structure of the RS-14 (Surzykiewicz et al., 2019). The reliability of the scale, calculated using Cronbach’s α coefficient, was *α* = 0.85 for the entire sample.

#### Religious coping

To measure religious coping we used the Polish version of the Religious Coping Questionnaire RCOPE (Pargament et al., 2000), which was adopted by Talik and Szewczyk (2008). The religious strategies under consideration represent a broad spectrum of the studied reality—they include positive and negative, passive, active, and interactive strategies relating to God and the Church. The complexity and multidimensionality of the phenomenon have been confirmed by the results of factor analysis. The Polish version of the RCOPE consists of 105 items on 16 scales, with both positive (9) and negative (7) religious strategies, but only 85 items are diagnostic. People respond to the items on a 4-point Likert scale, with their answers ranging from 0, “not at all” to 3, “a great deal”. They assess the degree to which they make use of various religious coping strategies (positive: *α* = 0.91 and negative: *α* = 0.71). The scores are calculated from the 16 scales (Talik, 2009, 2011, 2013; Talik and Szewczyk, 2008). The positive religious strategies are as follows: Life Transformation (LT)—looking to religion for a radical change in life; seeking a new direction for life, *α* = 0.92; Active Religious Surrender (ARS)—voluntarily giving up control to God in order to cope, *α* = 0.89; Seeking Support from Priests/Members (SSM)—searching for comfort, prayers and spiritual support from priests or church members, *α* = 0.84; Religious Focus (RF)—engaging in religious activities to shift the focus from the stressor, *α* = 0.85; Collaborative Religious Coping (CRC)—partnership with God in problem solving, and redefining the stressor through religion as benevolent, *α* = 0.86; Pleading for Direct Intercession (PDI)—pleading to God for a miracle or divine intercession, *α* = 0.78; Spiritual Support (SS)—seeking and giving spiritual support to others, *α* = 0.82; Religious Practices (RP)—active practising of and faithfulness to religious principles, *α* = 0.86; and Benevolent Religious Reappraisal (BRR)—redefining the stressor through religion as benevolent and potentially beneficial, *α* = 0.85. The negative religious strategies are as follows: Punishing God Reappraisal (PGR)—redefining the stressor as a punishment from God for the individual’s sins, *α* = 0.86; Self-directing Religious Coping (SRC)—coping without God’s help, *α* = 0.83; Demonic Reappraisal (DR)—redefining the stressor as the act of the devil, *α* = 0.99; Passive Religious Deferral (PRD)—passive waiting for God to control the situation and solve the problem, *α* = 0.85; Spiritual Discontent (SD)expressing dissatisfaction and anger about God’s relationship with the individual in a stressful situation, *α* = 0.84; Reappraisal of God’s Power (RGP)—questioning God’s power to influence the stressful situation, *α* = 0.66; and Religious Discontent (RD)—dissatisfaction with congregation members and questioning the Church’s teaching, *α* = 0.71 (Talik and Szewczyk, 2008).

#### Mental well-being

The Warwick-Edinburgh Mental Well-being Scale (WEMWBS) was applied to measure mental well-being (Stewart-Brown et al., 2011). In the view of the authors, psychological well-being covers the hedonistic and eudaimonic dimensions. States of happiness and satisfaction with life, positive psychological functioning, good relationships with others and self-realization/acceptance (Tennant et al., 2007; Stewart-Brown and Janmohamed, 2008). Participants were asked to respond to 14 items on a 5-point Likert scale: from 1 (“none of the time”) to 5 (“all of the time”). The distribution of points within the WEMWBS scale is within the area of 14–70. Polish adaptation studies validated the original one-dimensional structure of the WEMWBS (Konaszewski et al., 2021b). The reliability of the scale, calculated with Cronbach’s α coefficient, turned out to be high and amounted to *α* = 0.92 across the entire sample.

### Data analysis

An *a priori* G*Power 3.1. analysis was conducted to determine the suitable sample size. We used the suggested higher power criteria of 0.95 and a critical significance level of α of 0.05 to identify a medium effect size of *f*^2^ = 0.15. The total number of variables is 18. G*Power analysis with the above-mentioned parameters would demand a sample of at least 208 participants. Pearson’s correlation analysis was used to determine the relations between the variables. The mediation model was assessed using Hayes’ Process macro. The significance level was determined at *p* < 0.050. Furthermore, the false discovery rate (FDR; Benjamini–Hochberg correction) method was used to minimalize the risk of making a type I error in multiple comparisons. The effect size was assessed based on *R*^2^. Data analysis was conducted in IBM SPSS Statistics 26. The PROCESS macro in version 3.2 (Hayes, 2013) was applied to control whether the dimensions of RCOPE would mediate the relationship between trait resilience and mental well-being. Trait resilience acted as the independent variable and mental well-being as the dependent variable. Life Transformation, Active Religious Surrender, Seeking Support from Priests/Members, Religious Focus, Collaborative Religious Coping, Pleading for Direct Intercession, Spiritual Support, Religious Practices, Benevolent Religious Reappraisal, Punishing God Reappraisal, Self-directing Religious Coping, Demonic Reappraisal, Passive Religious Deferral, Spiritual Discontent, Reappraisal of God’s Power and Religious Discontent were treated as mediating variables (separately). Consequently, we analyzed 14 single-level mediation models (Model no. 4), comprising three-variable systems. Age and sex were considered as potential covariates in all models and were retained if significantly related to mental well-being. The bootstrap estimates and 95% confidence intervals (CI) for the indirect effects were gained through the procedure of 5,000 bootstrapped samples.

## Results

The average value of overall positive religious coping (*M* = 76.59; *SD* = 26.12) indicated a high degree of this type of coping within the studied group (seventh sten; Talik, 2013). On the other hand, the value of the mean of general negative religious coping (*M* = 29.74; *SD* = 14.78) demonstrated the average intensity of this variable within the studied group (sixth sten; Talik, 2013). As far as positive religious coping strategies go, the lowest values were recorded for seeking support from priests/members (*M* = 6.09, *SD* = 3.90), and the highest were recorded for religious practices (*M* = 15.08; *SD* = 4.33). In turn, in the group of negative strategies, the respondents rarely applied the religious discontent strategy (*M* = 2.27, *SD* = 1.34), with the most popular being self-directing religious coping (*M* = 5.95, *SD* = 2.94). The remaining descriptive results, as well as the results of the correlation analysis, are presented in Table 1.

The correlation analysis demonstrated that resilience significantly and positively combines with mental well-being and individual strategies of positive copings, such as active religious surrender, collaborative religious coping, spiritual support and religious practice. The relationship between resilience and positive coping was irrelevant. On the other hand, there was a significant relationship between resilience and the total score of negative religious coping. We also noticed significant negative relationships between resilience and punishing god reappraisal, demonic reappraisal, passive religious deferral, spiritual discontent and religious discontent. The relationships between negative and positive religious coping with mental well-being were irrelevant. Within the group of positive strategies of religious coping, three significant and positive associations with mental well-being were noted: collaborative religious coping, spiritual support and religious practices, whereas the group of negative strategies contained only two significant associations with mental well-being. The punishing god reappraisal and spiritual discontent strategies were connected negatively with mental well-being. The values of correlation coefficients are presented in Table 2; the analysis also takes into account the correlations between the individual religious coping strategies.

**Table 2:** Descriptive statistics and correlations (*N* = 317).

Min Max			M (SD)	RE	WB	LT	ARS	SSM	RF	CRC	PDI	SS	RP	BRR	PGR	SRC	DR	PRD	SD	RGP	PRC	RD
**RE**	21	98	74.63 (12.99)	1				
**WB**	14	70	52.41 (9.68)	**0.67** ^***^	1				
**LT**	0	21	10.09 (5.39)	−0.04	−0.03	1			
**ARS**	0	15	8.68 (3.71)	**0.12** ^*^	0.04	**0.56** ^***^	1											
**SSM**	0	15	6.09 (3.90)	0.04	0.09	**0.66** ^***^	**0.50** ^***^	1									
**RF**	0	15	6.56 (3.56)	0.01	0.02	**0.54** ^***^	**0.52** ^***^	**0.55** ^***^	1						
**CRC**	0	15	9.07 (3.12)	**0.19** ^***^	**0.14** ^*^	**0.51** ^***^	**0.68** ^***^	**0.50** ^***^	**0.51** ^***^	1						
**PDI**	0	12	7.08 (2.52)	0.05	0.08	**0.53** ^***^	**0.59** ^***^	**0.47** ^***^	**0.61** ^***^	**0.60** ^***^	1							
**SS**	0	15	6.99 (3.63)	**0.14** ^*^	**0.15** ^**^	**0.71** ^***^	**0.56** ^***^	**0.76** ^***^	**0.58** ^***^	**0.55** ^***^	**0.53** ^***^	1			
**RP**	0	24	15.08 (4.33)	**0.18** ^***^	**0.24** ^***^	**0.61** ^***^	**0.58** ^***^	**0.57** ^***^	**0.48** ^***^	**0.65** ^***^	**0.52** ^***^	**0.64** ^***^	1				
**BRR**	0	12	6.95 (2.78)	0.06	0.06	**0.59** ^***^	**0.62** ^***^	**0.53** ^***^	**0.58** ^***^	**0.60** ^***^	**0.52** ^***^	**0.61** ^***^	**0.63** ^***^	1		
**PGR**	0	15	5.32 (3.61)	**−0.26** ^***^	**−0.13** ^*^	**0.34** ^***^	**0.12** ^*^	**0.19** ^***^	**0.39** ^***^	0.05	**0.38** ^***^	**0.16** ^**^	0.08	**0.15** ^**^	1			
**SRC**	0	15	5.95 (2.94)	−0.06	0.08	**−0.22** ^***^	**−0.31** ^***^	**−0.16** ^**^	−0.07	**−0.40** ^***^	**−0.22** ^***^	**−0.20** ^***^	**−0.25** ^***^	**−0.28** ^***^	**0.20** ^***^	1			
**DR**	0	12	3.32 (2.88)	**−0.21** ^***^	−0.04	**0.42** ^***^	**0.18** ^***^	**0.43** ^***^	**0.50** ^***^	**0.22** ^***^	**0.32** ^***^	**0.39** ^***^	**0.25** ^***^	**0.26** ^***^	**0.52** ^***^	0.07	1	
**PRD**	0	13	4.42 (3.36)	**−0.25** ^***^	−0.06	**0.51** ^***^	**0.26** ^***^	**0.56** ^***^	**0.53** ^***^	**0.25** ^***^	**0.38** ^***^	**0.45** ^***^	**0.28** ^***^	**0.37** ^***^	**0.43** ^***^	0.09	**0.59** ^***^	1		
**SD**	0	16	4.54 (3.91)	**−0.32** ^***^	**−0.13** ^*^	**0.43** ^***^	0.10	**0.33** ^***^	**0.34** ^***^	−0.06	**0.24** ^***^	**0.28** ^***^	0.05	**0.14** ^*^	**0.64** ^***^	**0.31** ^***^	**0.55** ^***^	**0.56** ^***^	1	
**RGP**	0	12	3.93 (2.74)	−0.09	0.07	**0.18** ^***^	0.10	**0.23** ^***^	**0.24** ^***^	−0.01	**0.26** ^***^	**0.12** ^*^	0.05	0.03	**0.51** ^***^	**0.41** ^***^	**0.37** ^***^	**0.40** ^***^	**0.58** ^***^	1	
**RD**	0	6	2.27 (1.34)	**−0.19** ^***^	−0.09	**0.13** ^*^	−0.10	0.03	0.02	**−0.16** ^**^	0.05	0.08	−0.04	−0.05	**0.33** ^***^	**0.36** ^***^	**0.22** ^***^	**0.24** ^***^	**0.54** ^***^	**0.34** ^***^	
**PRC**	9	138	76.59 (26.12)	0.09	0.11	**0.83** ^***^	**0.78** ^***^	**0.79** ^***^	**0.74** ^***^	**0.77** ^***^	**0.73** ^***^	**0.85** ^***^	**0.81** ^***^	**0.79** ^***^	**0.26** ^***^	**−0.29** ^***^	**0.43** ^***^	**0.51** ^***^	**0.28** ^***^	**0.17** ^**^	1
**NRC**	2	79	29.74 (14.78)	**−0.29** ^***^	−0.07	**0.40** ^***^	0.09	**0.36** ^***^	**0.43** ^***^	0.01	**0.31** ^***^	**0.28** ^***^	0.09	**0.16** ^**^	**0.78** ^***^	**0.47** ^***^	**0.71** ^***^	**0.71** ^***^	**0.87** ^***^	**0.74** ^***^	**0.31** ^***^	**0.55** ^***^

*p < 0.05; **p < 0.01; ***p < 0.001; RE, resilience; WB, mental well-being; LT, Life Transformation; ARS, Active Religious Surrender; SSM, Seeking Support from Priests/Members; RF, Religious Focus; CRC, Collaborative Religious Coping; PDI, Pleading for direct Intercession; SS, Spiritual Support; RP, Religious Practices; BRR, Benevolent Religious Reappraisal; PGR, Punishing God Reappraisal; SRC, Self-directing Religious Coping; DR, Demonic Reappraisal; PRD, Passive Religious Deferral; SD, Spiritual Discontent; RGP, Reappraisal of God’s Power; RD, Religious Discontent; PRC, total score of Positive Religious Coping; NRC, total score of Negative Religious Coping.

**Figure 1:**
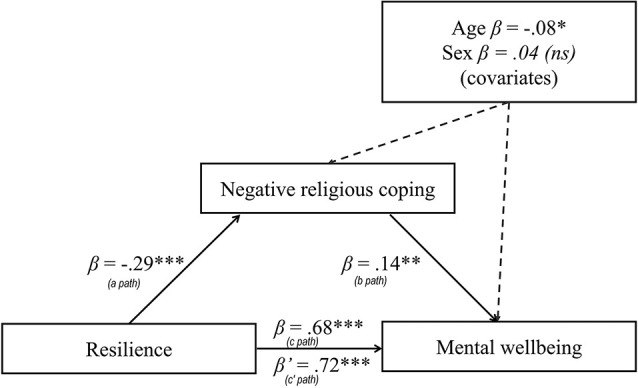
Negative religious coping as mediator in relationship between resilience and mental well-being (age and sex as covariates).

Bootstrap sampling analysis (5,000) with 95% confidence intervals displayed several significant partial mediators for the relationship between resilience and mental well-being. An important mediator was the total score of negative religious coping, three detailed strategies of negative coping (demonic reappraisal, passive religious deferral, spiritual discontent) and one strategy of positive religious coping (religious practices). The total effect (*c path*) amounted to *β* = 0.68 (*t* = 16.35, *p* < 0.001; *R*^2^ = 0.46). In the case of the total score of negative religious coping, the regression coefficient of the independent variable on the mediator (*a path*) amounted to *β* = −0.29 (*t* = −5.36, *p* < 0.001; *R*^2^ = 0.10). The mediator regression coefficient on the dependent variable with simultaneous control of the independent variable (*b path*) amounted to *β* = 0.14 (*t* = 3.17, *p* = 0.002; *R*^2^ for the entire model = 0.48). Mediation increased the strength of the relationship between resilience and mental well-being in a direct effect (*c^′^ path*) amounted to *β* = 0.72 (*t* = 16.80, *p* < 0.001). Figure 1 shows the relationship between resilience and mental well-being with negative religious coping as a mediator (age and sex as covariates).

The *a path* for demonic reappraisal totaled *β* = −0.21 (*t* = −3.85, *p* < 0.001; *R*^2^ = 0.04), *b path* totaled *β* = 0.11 (*t* = 2.66, *p* = 0.008; *R*^2^ for the entire model = 0.47) with the * c^′^ path* totaling *β* = 0.70 (*t* = 16.70, *p* < 0.001). The *a path* for passive religious deferral totaled *β* = −0.25 (*t* = −4.52, *p* < 0.001; *R*^2^ = 0.06), the *b path* totaled *β* = 0.11 (*t* = 2.48, *p* = 0.014; *R*^2^ for the entire model = 0.47) and the *c^′^ path* totaled *β* = 0.70 (*t* = 16.59, *p* < 0.001). The *a path* for spiritual discontent totaled *β* = −0.32 (*t* = −5.92, *p* < 0.001; *R*^2^ = 0.10), the *b path* totaled *β* = 0.10 (*t* = 2.26, *p* = 0.025; Benjamini–Hochberg adjusted *p-*value = 0.062; *R*^2^ for the entire model = 0.47) and the *c^′^ path* totaled *β* = 0.71 (*t* = 16.33, *p* < 0.001). For the effects described, when we include the mediating variable, the relationship between resilience and mental well-being is intensified. This indicates the presence of suppression. In the remaining cases, negative forms of religious coping did not mediate the relationship between resilience and mental well-being in a statistically significant manner. General positive religious coping did not mediate the relationship between resilience and mental well-being. Taking into account the detailed strategies, one statistically significant mediation effect was obtained by religious practices, i.e., the *a path* totaled *β* = 0.18 (*t* = 3.25, *p* = 0.001; *R*^2^ = 0.03), the *b path* totaled *β* = 0.12 (*t* = 2.80, *p* = 0.006; *R*^2^ for the entire model = 0.48), and the *c^′^ path* totaled *β* = 0.66 (*t* = 15.74, *p* < 0.001). The other particular positive religious coping strategies did not mediate the relationship between resilience and mental well-being. The significance level for the effects is presented in Table 3.

**Table 3:** The role of religious coping strategies on resilience and mental well-being (*N* = 317).

	a Path	b Path	c Path	c^′^Path	Indirect effect and B (SE)	95% CI Lower upper
**Positive religious coping**						
RE → LT → WB	−0.04	−0.01	0.68***	0.68^***^	0.0001 (0.0029)	−0.0057; 0.0070
RE → ARS → WB	0.12**	−0.04	0.68^***^	0.68^***^	−0.0055 (0.0061)	−0.0195; 0.0056
RE → SSM → WB	0.04	0.07	0.68***	0.67^***^	0.0029 (0.0045)	−0.0048; 0.0135
RE → RF → WB	0.01	0.01	0.68***	0.68^***^	0.0001 (0.0024)	−0.0048; 0.0056
RE → CRC → WB	0.19***	0.01	0.68^***^	0.68^***^	0.0025 (0.0086)	−0.0140; 0.0214
RE → PDI → WB	0.05	0.05	0.68***	0.68^***^	0.0022 (0.0044)	−0.0053; 0.0130
RE → SS → WB	0.14**	0.05	0.68^***^	0.67^***^	0.0069 (0.0070)	−0.0043; 0.0237
**RE → RP → WB**	**0.18** ^**^		**0.68** ^***^	**0.66** ^***^	**0.0212 (0.0119)**	**0.0019; 0.0475**
RE → BRR → WB	0.06	0.02	0.68***	0.68^***^	0.0011 (0.0040)	−0.0047; 0.0118
RE → PRC → WB	0.09	0.04	0.68***	0.68^***^	0.0038 (0.0054)	−0.0048; 0.0173
**Negative religious coping**						
RE → PGR → WB	−0.26***	0.05	0.68^***^	69^***^	−0.0116 (0.0128)	−0.0406; 0.0106
RE → SRC → WB	−0.06	0.11**	0.68^***^	0.68^***^	−0.0059 (0.0073)	−0.0227; 0.0072
**RE → DR → WB**	**−0.21** ^***^	**0.11** ^**^	**0.68** ^***^	**70** ^***^	**−0.0238 (0.0120)**	**−0.0510; −0.0044**
**RE → PRD → WB**	**−0.25** ^***^	**0.11** ^**^	**0.68** ^***^	**70** ^***^	**−0.0257 (0.0129)**	**−0.0551; −0.0045**
**RE → SD → WB**	**−0.32** ^***^	**0.10** ^*^ *^a^*	**0.68** ^***^	**71** ^***^	**−0.0309 (0.0171)**	**−0.0663; −0.0004**
RE → RGP → WB	−0.09	0.13**	0.68^***^	0.69^***^	−0.0111 (0.0090)	−0.0313; 0.0035
RE → RD → WB	−0.19***	0.03	0.68^***^	0.68^***^	−0.0047 (0.0090)	−0.0243; 0.0125
**RE → NRC → WB**	**−0.29** ^***^	**0.14** ^**^	**0.68** ^***^	**72** ^***^	**−0.0389 (0.0163)**	**−0.0744; −0.0111**

*p < 0.05; **p < 0.01; ***p < 0.001; ^a^not significant using an FDR of 0.05; RE, resilience; WB, mental well-being; LT, Life Transformation; ARS, Active Religious Surrender; SSM, Seeking Support from Priests/Members; RF, Religious Focus; CRC, Collaborative Religious Coping; PDI, Pleading for direct Intercession; SS, Spiritual Support; RP, Religious Practices; BRR, Benevolent Religious Reappraisal; PRC, total score of Positive Religious Coping; PGR, Punishing God Reappraisal; SRC, Self-directing Religious Coping; DR, Demonic Reappraisal; PRD, Passive Religious Deferral; SD, Spiritual Discontent; RGP, Reappraisal of God’s Power; RD, Religious Discontent; NRC, total score of Negative Religious Coping; a path, effect of the independent variable on the mediator; b path, effect of the mediator on the dependent variable; c path, effect of the independent variable on the dependent variable; c′ path, direct effect of the independent variable on the dependent variable while controlling for the mediator. Effects are adjusted for age and sex.

## Discussion

The purpose of the study was to examine the relationship between resilience and mental well-being. We extended the scope of analysis and examined both direct and indirect relationships between these variables. Similarly, like our predecessors, we focused on mediating role of stress-related variables and examined the mediating role of religious coping. That mechanism has not been explored yet, to the best of our knowledge. The aim of this study was to demonstrate the mediating role of positive and negative religious coping in the relationship between the trait of resilience and mental well-being. Our study analyzed both the mediating role of both types of religious coping, as well as particular strategies in the group of practising Catholics. As Pargament (1992) points out, ways of religious coping are interrelated; when faced with difficult situations, individuals may apply many positive and negative strategies. The results of our research indicate that the respondents when in difficult situations, used positive religious coping more willingly than negative coping. The average value of religious coping indicated a high peak in positive coping and moderate results in the case of negative coping. The most frequently used positive strategy was religious practices, and the least frequent was seeking support from priests/members. High scores in religious practices might be considered not only as a useful coping strategy but also as an expression of being religious for Polish Catholics. Low scores on seeking support from priests may be related to restricted immediate access to them during the COVID-19 pandemic. In turn, self-directing religious coping was the strategy most often used in the group of negative strategies, with religious discontent as the least frequent. A clear propensity to use positive strategies can be seen as a personal resource for practising Catholics. On the other hand, the use of negative religious coping strategies can be linked with a relatively stable tendency to experience tensions related to matters of faith and one’s relationship with God (Zarzycka, 2017). It should also be taken into account that negative religious coping can be triggered when the level of difficulties experienced by the individual is so great that it exceeds the resources that were sufficient to deal with everyday problems (Hreciński, 2017).

### Relationships between resilience, religious coping, and mental well-being

The obtained results displayed a significant relationship between resilience and mental well-being. Similarly to the studies of our predecessors, our research also demonstrated that the higher the intensity of resilience, the higher the level of mental well-being (Abolghasemi and Varaniyab, 2010; Ya et al., 2012; He et al., 2013; Liu et al., 2013; Aiena et al., 2015; Di Fabio and Palazzeschi, 2015; Smith and Hollinger-Smith, 2015; Surzykiewicz et al., 2019; Konaszewski et al., 2021a). The relationship between resilience and general negative religious coping was also important but low. The results confirmed the negative nature of this relationship (McIntire and Duncan, 2013; Rezapur-Shahkolai et al., 2017; Konaszewski et al., 2020). The results obtained regarding the relationship between resilience and negative religious coping are consistent with previous research that has shown that the higher the level of resilience, the lower the tendency to use strategies focused on negative emotions and the need to discharge them (Boyden and Mann, 2005; Campbell-Sills et al., 2006; Chen, 2016; Konaszewski et al., 2019). Thus, it can be concluded that resilience is related to the tendency to view difficult events in terms of God’s punishment, religious passivity, or experiencing dissatisfaction with God and the church. In other words, the tendency to use negative religious coping is associated with reduced levels of resilience resources and therefore is consistent with previous research (Pargament et al., 1990, 2000, 2001; Fallot and Heckman, 2005). To perceive God as a punisher, to experience abandonment by God, and “spiritually desert” experiences closely relate to phases of spiritual dryness (Büssing et al., 2013, 2021). Research demonstrates that feelings of spiritual dryness are associated with perceived stress, depression, anxiety, and emotional exhaustion (Büssing et al., 2013). Experiencing spiritual dryness is also linked to poor mental well-being. A study conducted during the COVID-19 pandemic among Iranian Muslims showed that spiritual dryness is moderately related to lower life satisfaction and marginally related to poor well-being (Büssing et al., 2021). Furthermore, spiritual dryness can lead to either a loss of faith or spiritual growth (Büssing et al., 2020).

In the study the relationship between resilience and general positive religious coping was irrelevant. Similar relationships were established, among others, in studies by Jans-Beken (2019) and Konaszewski et al. (2020). The lack of relationship between the variables may be due to the fact that resilience represents an active, problem-solving approach to stressful situations, while positive religious coping represents a more passive, avoidance-oriented approach, such as ‘focusing on religion to stop worrying about my problems, or “surrendering to God’s will.”’ This does not imply that positive religious coping only includes an avoidant approach to difficult situations.

Although the relationship between overall negative religious coping and mental well-being was negligible, we found a significant and negative relationship between mental well-being and two component strategies: punishing god reappraisal and spiritual discontent. And so, the results of our study have not confirmed the findings existing so far, indicating that negative religious coping is generally associated with decreased well-being (Pargament et al., 2001; Wnuk, 2007; Hebert et al., 2009; Scandrett and Mitchell, 2009; Krok, 2014; Taheri-Kharameh et al., 2016). Although the mechanisms underlying the described relationships have not been clearly established, one possibility is that punishing God’s reappraisal and spiritual discontent may decrease well-being by reducing people’s efforts to stay healthy while serving as a trigger for risky behaviors. Park et al. (2009) showed that negative religious coping was associated with lower medication adherence and medication use, as well as higher levels of alcohol use among patients. In addition, although some individuals agree with the belief that God is forgiving, at the same time their personal experiences are associated with a sense of unforgiveness on the part of God. Feeling unforgiveness triggers fear of God’s punishment, which results in reduced well-being (Zarzycka, 2017). Also, experiencing doubt in terms of faith is not foreign to members of religious communities (Krause et al., 1999). It can be inferred that struggles involving spiritual discontent are associated with poorer psychological functioning. This relationship pattern has been repeated in many studies, and as the results of longitudinal studies suggest that this relationship may weaken over time (Zarzycka, 2017). Also in the case of overall positive coping, we have not noticed any significant relationship between this variable and mental well-being. Only three strategies (collaborative religious coping, spiritual support and religious practices) have shown positive relationships with mental well-being. The results of our studies indicate that the holistic concept of mental well-being may not prove helpful in the analysis of the relationship with religious coping, due to its hedonistic dimension, which focused more on seeking pleasure and positive experiences and high satisfaction with life than on the individual’s involvement in dealing with existential challenges posed by life (Keyes et al., 2002).

### The mediating effects of negative and positive religious coping between resilience and mental well-being

The analysis of mediation has demonstrated that taking into account within the model of the general negative religious coping and the strategies of demonic reappraisal, passive religious deferral, and spiritual discontent have intensified the positive relationship between resilience and mental well-being, which point to the appearance of the suppression phenomenon. We controlled age and sex as statistically significant covariates in our analyzes, so that the mediation effects obtained were devoid of the influence of those critical variables on the models. It is a well-established fact that religious struggle and negative coping can harm mental health (Ellison et al., 2010; Exline, 2013; Wilt et al., 2016), although studies by Zarzycka and Zietek (2019) demonstrated that demonic and moral religious struggles can promote well-being, as they can be a source of positive changes and lead to spiritual growth. Demonic re-evaluation means accepting a stressful situation as Satan’s work. More specifically, demonic evaluation is a belief that the devil or other demonic forces have a direct or indirect influence on someone or something (Talik and Szewczyk, 2008). The ability to attribute negative events to evil forces may facilitate some people in gaining a sense of support from their faith, as it allows them to protect their image of God and their relationship with God (Zarzycka and Zietek, 2019).

Religious passivity and dissatisfaction with God also enhance the effect that resilience has on well-being. Research on the function of faith can be helpful in understanding this result. Many people firmly believe in a passive attitude, in which expectations are emphasized for God to take control of the situation; shifting responsibility for solving the problem to God will help in a stressful situation. On the other hand, expressing dissatisfaction and anger mainly towards the attitude of God and the Church towards a person in a stressful situation (feelings of abandonment, rejection, of being unloved) may lead to the mobilization of forces helpful in difficult situations. It is consistent with the idea that religion offers individuals several ways to maintain and increase their sense of well-being and the possibility of spiritual growth and development (Spilka et al., 1985). This perspective, present in the work by Batson et al. (1993) on religious exploration, is based in part on the assumption that religious doubt is beneficial and leads to a deeper and more meaningful faith. Also, Tillich (1957) argues that various strategies and struggles that may be described as negative are not inherently wrong. Doubt and dissatisfaction are not the opposite of faith; rather, they are part of it.

Research results suggest that, in the case of well-being, negative strategies should be ascribed significant importance. It can be concluded that general negatively focused strategies, including demonic reappraisal, passive religious deferral, and spiritual discontent are an important part of faith (Hunsberger et al., 1996, 2002; Kooistra and Pargament, 1999). It would be difficult to imagine a deeply religious person who has no moments of doubt, dissatisfaction, or passivity about their religious beliefs. This struggle can be considered an effort to preserve or transform an endangered spirituality. The struggles themselves may focus on the expression of suffering, anger, dissatisfaction, fear, and disorientation (Zarzycka, 2017). While numerous studies confirm the negative relationship between religious coping and well-being, quality of life and health studies also exist that have not confirmed it (Hunsberger et al., 2002), while still others have found positive relationships (Krause and Wulff, 2004; Exline et al., 2014). Faced with such inconsistent data, it is legitimate to ask about the mechanisms by which we can explain the relationships between resilience, coping strategies, and well-being. We have come to the conclusion that the impact of resilience on mental well-being, through negative religious coping strategies, depends on how we treat them. This is demonstrated by some psychological theories, for example by Erikson, Kohlberg, or Dąbrowski, focusing on positive disintegration; the function of moral conflicts or crises inherent in development. They are a transitional phase that can lead to both regression as well as maturity and increased well-being (e.g., Erikson, 1968; Kohlberg, 1976; Dąbrowski, 1979). Our findings suggest that resilience increases well-being through negative coping, considering low negative coping as an immediate effect (a reactive process). This finding confirms that demonic reappraisal, passive religious deferral, and spiritual discontent can be part of a healthy process and suggests that negative coping enhances the relationship between resilience and mental well-being (Batson and Schoenrade, 1991; Exline et al., 2014; Zarzycka and Zietek, 2019).

Our analysis found that overall positive religious coping did not mediate the relationship between resilience and mental well-being. After taking detailed strategies into account, one statistically significant mediation effect was obtained by religious practices. Faithfulness to religious practices involves actively practising and faithfully adhering to the teachings of one’s religion (steering away from false teachings). The relationship between resilience and mental well-being diminished when this coping strategy was taken into account. In the remaining cases, it was not noted that positive strategies mediate the relationship between resilience and mental well-being. This result is inconsistent with our expectations based on the theory and the results of previous studies (Pargament et al., 2001; Wnuk, 2007; Scandrett and Mitchell, 2009). In our study, we made the assumption that positive religious counseling should enhance the resilience effect on well-being within a group of practising Catholics. The observed lack of mediation effect can be explained by referring to the results of the meta-analysis by Ano and Vasconcelles (2005), which demonstrated that the relationship between positive religious coping and stress is noticeable only in the long term. The results of this study may therefore suggest that a positive appeal to religion protects against long-term consequences of difficulties. Undoubtedly, further exploration in this area is advisable.

Finally, it should be noted that the percentile bootstrap confidence intervals for indirect effects calculated by PROCESS are preferred for testing models with a large number of potential mediators and do not require additional adjustments (Taylor et al., 2008; Yzerbyt et al., 2018). Furthermore, in multiple comparisons, the authors suggest leaving a 95% confidence level for all confidence intervals in output at 5,000 number of bootstrap samples. On the other hand, because of the risk of making a Type I error, it is common to use corrections for multiple comparisons, which reduce the nominal significance level of each test by a specified correction. The disadvantage of such solutions is lowering the power of the test, i.e., increasing the risk of making a type II error. FDR is preferred in exploratory studies (Hochberg and Benjamini, 1990). After including this method in our study, the relationship between spiritual discontent and well-being while controlling for resilience proved to be statistically insignificant. The above indicates a questionable mediation effect. If so, this result should be interpreted with caution, and the analysis should be repeated in future studies without using multiple comparisons. The Benjamini-Hochberg correction did not affect the other revealed relationship effects.

### Practical implications

Our findings offer indications for the development of resilience- and coping-based interventions to protect the mental health and well-being of individuals. The results of this study generally indicate that the development of psychological resources is able to help protect mental health from negative coping. Resources such as resilience can be increased through psychological intervention and treatment programs (Ritchie et al., 2014).

### Limitations

In our study, we have focused on the mediating role of religious coping in the relationship between resilience and mental well-being. For the purposes of further research, an attempt should be made to verify the mediating role of religious struggles and tensions. It is also worth taking into account the level of religiosity and religious centrality in the analyzes, as both of these factors can be potential moderators of the relationship between resilience and well-being. Our study has its limitations. The main limitation of the study is its transverse character, excluding any conclusions regarding the cause-and-effect relationships. Mediation analysis is able to test the significance and perhaps the effect size of a hypothetical mediator, assuming it is the actual mediator. On the other hand, mediation analysis cannot determine whether a variable is a unique (sole) mediator, and significant mediation tests do not provide sufficient evidence to support the causal role played by the intervening variable (mediator) in the model being tested. Longitudinal studies are necessary to assess resilience and the religious coping function of well-being, health, and overall frame of mind. Another limitation is related to the time period in which the study was conducted and the characteristics of the studied sample. The data were collected during the second wave of the COVID-19 pandemic and this may have influenced the findings. Moreover, the participants of our study were people aged 19–60 years, but with a clear predominance of young people. Research demonstrates that the depth of religiosity changes with age, and therefore it can be observed that positive religious coping is more often used by older people (Pargament, 1997). Therefore, further research should consider a moderated mediation analysis to compare the relationship between resilience, religious coping, and well-being among younger and older (over 35 years of age) individuals due to the greater religiosity of the latter.

## Conclusion

This is the first study to investigate the role of religious coping as a mediator in the relationship between resilience and mental well-being. The obtained results demonstrate the occurrence of the suppression effect in the case of general negative religious coping and its three components. Negative religious coping, demonic reappraisal, passive religious deferral, and spiritual discontent enhance the relationship between resilience and mental well-being. Research has demonstrated no significant mediation effect on overall positive religious coping. Among the components of overall positive coping, only one strategy, i.e., religious practices, had a significant effect. The effect of resilience on mental well-being was diminished by the use of religious practices.

## Data Availability Statement

The raw data supporting the conclusions of this article will be made available by the authors, without undue reservation.

## Ethics Statement

The studies involving human participants were reviewed and approved by the Ethics Committee of the Faculty of Education of the University of Białystok in Białystok. The patients/participants provided their written informed consent to participate in this study.

## Author Contributions

KK, SS, MN, and JS: methodology, formal analysis, writing—original draft preparation, writing—review and editing. KK: investigation. JS, KK, and MN: project administration and funding acquisition. All authors contributed to the article and approved the submitted version.

## Conflict of Interest

The authors declare that the research was conducted in the absence of any commercial or financial relationships that could be construed as a potential conflict of interest.

## Publisher’s Note

All claims expressed in this article are solely those of the authors and do not necessarily represent those of their affiliated organizations, or those of the publisher, the editors and the reviewers. Any product that may be evaluated in this article, or claim that may be made by its manufacturer, is not guaranteed or endorsed by the publisher.
